# Frontal mechanisms underlying primate calls recognition by humans

**DOI:** 10.1093/texcom/tgad019

**Published:** 2023-11-02

**Authors:** Leonardo Ceravolo, Coralie Debracque, Eva Pool, Thibaud Gruber, Didier Grandjean

**Affiliations:** Neuroscience of Emotions and Affective Dynamics lab, Department of Psychology and Educational Sciences, University of Geneva, Unimail building, Boulevard Pont-d’Arve 40CH-1205, Geneva, Switzerland; Swiss Center for Affective Sciences, University of Geneva, Campus Biotech building, Chemin des Mines 9CH-1202, Geneva, Switzerland; Neuroscience of Emotions and Affective Dynamics lab, Department of Psychology and Educational Sciences, University of Geneva, Unimail building, Boulevard Pont-d’Arve 40CH-1205, Geneva, Switzerland; Swiss Center for Affective Sciences, University of Geneva, Campus Biotech building, Chemin des Mines 9CH-1202, Geneva, Switzerland; Swiss Center for Affective Sciences, University of Geneva, Campus Biotech building, Chemin des Mines 9CH-1202, Geneva, Switzerland; E3 Lab, Department of Psychology and Educational Sciences, University of Geneva, Unimail building, Boulevard Pont-d’Arve 40CH-1205, Geneva, Switzerland; Neuroscience of Emotions and Affective Dynamics lab, Department of Psychology and Educational Sciences, University of Geneva, Unimail building, Boulevard Pont-d’Arve 40CH-1205, Geneva, Switzerland; Swiss Center for Affective Sciences, University of Geneva, Campus Biotech building, Chemin des Mines 9CH-1202, Geneva, Switzerland; eccePAN lab, Department of Psychology and Educational Sciences, University of Geneva, Campus Biotech building, Chemin des Mines 9CH-1202, Geneva, Switzerland; Neuroscience of Emotions and Affective Dynamics lab, Department of Psychology and Educational Sciences, University of Geneva, Unimail building, Boulevard Pont-d’Arve 40CH-1205, Geneva, Switzerland; Swiss Center for Affective Sciences, University of Geneva, Campus Biotech building, Chemin des Mines 9CH-1202, Geneva, Switzerland

**Keywords:** neuroimaging, decision-making, model-based, primate vocalizations, human brain

## Abstract

**Introduction:**

The ability to process verbal language seems unique to humans and relies not only on semantics but on other forms of communication such as affective vocalizations, that we share with other primate species—particularly great apes (*Hominidae*).

**Methods:**

To better understand these processes at the behavioral and brain level, we asked human participants to categorize vocalizations of four primate species including human, great apes (chimpanzee and bonobo), and monkey (rhesus macaque) during MRI acquisition.

**Results:**

Classification was above chance level for all species but bonobo vocalizations. Imaging analyses were computed using a participant-specific, trial-by-trial fitted probability categorization value in a model-based style of data analysis. Model-based analyses revealed the implication of the bilateral orbitofrontal cortex and inferior frontal gyrus *pars triangularis* (IFG_tri_) respectively correlating and anti-correlating with the fitted probability of accurate species classification. Further conjunction analyses revealed enhanced activity in a sub-area of the left IFG_tri_ specifically for the accurate classification of chimpanzee calls compared to human voices.

**Discussion:**

Our data—that are controlled for acoustic variability between species—therefore reveal distinct frontal mechanisms that shed light on how the human brain evolved to process vocal signals.

## Introduction

Processing and understanding non-verbal language are fundamental aspects of everyday life in human (Tracy et al. 2015) and animal (Marler 1967; Leger 1993) communication. Their importance can sometimes even be greater than that of verbal communication since a short, powerful in-context vocal burst—for instance, an urgency signal—can easily be noticed and understood by other individuals (Leger 1993). A critical part of non-verbal language can therefore be represented by “affective” communication, especially through affective vocalizations, which are of particular interest for cross-species research. Indeed, the ability to express and understand such vocalizations is shared by several primate species, including Hominids, a taxonomic family that includes humans and other great apes (Gagneux and Varki 2001; Prado-Martinez et al. 2013). Such commonalities therefore open a critical window into the investigation of human brain structures recruited by social communication, for instance by studying these crucial abilities to perceive, process and subsequently categorize and classify affective vocalizations expressed by primate species. These abilities are known to recruit superior temporal, orbitofrontal and inferior frontal cortices in humans (Binder et al. 2004; Murray and Izquierdo 2007; Frühholz et al. 2012; Grandjean 2020) while these brain areas evolved a lot in primates (Semendeferi et al. 2002; Damasio et al. 1993). Studying cross-species vocal signals processing and categorization taking place in the cortex of humans, especially the prefrontal cortex, is therefore of crucial interest in understanding social interactions.

The *Hominidae* clade appeared between 13 and 18 million years ago (Perelman et al. 2011). Encompassing all living great apes including humans (*Homo sapiens*), chimpanzees (*Pan troglodytes*), bonobos (*Pan Paniscus*)*,* gorillas (*Gorilla subs*), and orangutans (*Pongo subs*), as well as our extinct ancestors, this unique primate taxon is key to understanding the evolution of human behavior, physiology, communication and cognitive abilities. Very few studies have used bonobo vocalizations as stimuli (Kelly et al. 2017), despite sharing the same phylogenetic proximity to *H. sapiens* with chimpanzees, their behavior repertoire—of which gestures can be decoded by humans (Graham and Hobaiter 2023)—including their vocal communication are noticeably different (Hare et al. 2012; Gruber and Clay 2016; Grawunder et al. 2018; Staes et al. 2018). Despite a larger and more folded brain compared to other great ape species, human neuroanatomical traits are mainly considered to belong to a continuum in the primate brain evolution (Semendeferi et al. 2002; Herculano-Houzel 2009). For instance, findings in anatomical magnetic resonance imaging (MRI) have demonstrated the existence of a large frontal cortex in all great ape species—humans included (Semendeferi et al. 2002)—emphasizing the particular interest of investigating anatomical structures and related functions of the frontal regions. Moreover, the functions of the frontal lobe, often associated to problem solving, emotion processing and especially evaluative judgment, communication and language or even motor and sexual behaviors (Davidson 1992; Barbas 2000; Binder et al. 2004; Barbas et al. 2011; LeDoux 2012; Frühholz and Grandjean 2013; Kambara et al. 2018), as well as their related brain structures are shared by most primate species. This includes two critical brain structures that are the focus of this article: the orbitofrontal cortex (OFC) and the inferior frontal gyrus (IFG).

Existing even in phylogenetically distant non-human primate species such as rhesus macaques (*Macaca mulatta*) (Rolls 2004), the OFC has similar neuroanatomical architecture and functions across the primate family tree. From humans to monkeys, studies have highlighted the roles of OFC in emotional memory (Barbas 2000; Barbas et al. 2011), emotional expression (Sander et al. 2005; Murray and Izquierdo 2007), executive functions (Barbas 2000) and integration of contextual information in emotional evaluation (Grandjean 2020). Interestingly, despite these shared evolutionary OFC functions in primate species, its role in heterospecific affective recognition, such as affective vocalizations, has rarely been investigated. In fact, recent functional MRI data have pointed out a greater enhancement of activity in OFC, in particular of its medial part, in the human identification of emotional voices compared to affective animal vocalizations including positive and negative calls expressed by chimpanzees, macaques and cats (*Felis catus*) (Belin, Fecteau, et al. 2008a; Fritz et al. 2018). More investigations on the role of the human OFC in this context are thus crucially needed.

Functionally and anatomically connected to the OFC (Hsu et al. 2020), the IFG is also present in great apes as well as in monkey species (Petrides and Pandya 2002). Yet, unlike the OFC, the left IFG is most prominently linked to speech production (Broca’s area) in humans (Guenther et al. 2015), the only species “capable” of a complex and sophisticated language. Hence, in addition to likely shared functions such as decision formation (Binder et al. 2004; Brück et al. 2011), inhibition (Hampshire et al. 2010; Tops and Boksem 2011) and emotional judgment (Ethofer et al. 2006; Zhang et al. 2018; Grandjean 2020; Gruber et al. 2020) shaped by natural selection, the IFG also became specialized in semantic auditory processing in the human lineage in the course of evolution (Schirmer and Kotz 2006; Frühholz et al. 2012; Belyk et al. 2017). Because the current roles of human IFG are linked to both the *Hominidae* and the specific *H. sapiens* evolutionary histories (Stout and Chaminade 2012), this frontal region, together with the OFC, is of high interest to investigate conspecific and heterospecific vocalization identification mechanisms. Noteworthy is the fact that these specific frontal regions have strong anatomical ties with temporal lobe regions that are crucial in processing vocalizations, especially when they are biologically relevant (Ethofer et al. 2006; Ethofer et al. 2012; Frühholz and Grandjean 2013; Hsu et al. 2020). Besides the importance of OFC and IFG regions in the evolution of primate brains, comparative and integrative approaches to investigate the current functions of these specific frontal regions are still missing. This is especially true for auditory affective processing (Gruber and Grandjean 2017), species categorization and by extension non-verbal communication comprehension in humans—excluding sign language.

The present study therefore focused on the processing and subsequent categorization of primate vocalizations in human OFC and IFG using fMRI including four primate species (human non-verbal vocal stimuli, chimpanzee, bonobo, and macaque calls). Research on vocal production and perception has indeed demonstrated that non-verbal vocalizations expressed by humans are similar to those expressed by other mammalian species, non-human primates included, especially in affiliative (e.g. play) or agonistic contexts (e.g. aggression) (Anikin et al. 2018). Interestingly, as most animal vocalizations are expressed in a motivational or affective context, existing literature has remained focused on the human recognition of affects in heterospecific vocalizations while neglecting humans’ ability to identify other species “independently”—albeit true neutral expressions barely exist—of affect (Belin 2006; Scheumann et al. 2014; Filippi et al. 2017; Kelly et al. 2017; Scheumann et al. 2017; Fritz et al. 2018; Kamiloğlu et al. 2020). Moreover, the emphasis on the ability of modern humans, here our participants, to categorize primate vocalizations relies—using a parsimonious position—on either repeated encounter with primates, a hardwired mechanism that emerged through shared evolutionary history or through acoustic cues analysis. Recent work on this latter topic clarifies the role of acoustic distance on the evaluation of the selected primate species’ vocalizations (Debracque et al. 2023), and highlight the high probability that acoustic distance and phylogeny may well interact on this matter.

Hence, in light of previous literature as well as taking into account the commonalities and differences of the selected four primate species in their evolutionary path, we predicted: i) a behavioral gradient reflecting phylogenetic distance from humans for the species with a higher probability of correctly categorizing human, then chimpanzee, bonobo and finally macaque affective vocalizations (Scheumann et al. 2014; Filippi et al. 2017; Kelly et al. 2017); for similar reasons, we predicted that chimpanzee and bonobo vocalizations should be more confused with each other than with macaque calls; ii) neural correlates of the probability of correctly categorizing the four primate species in the OFC and IFG, especially in the medial OFC (Belin, Fecteau, et al. 2008a; Fritz et al. 2018) and IFG_tri/op_ (Dricu et al. 2017), respectively; iii) a gradient of activity following phylogeny in the *pars triangularis* and *opercularis* of the IFG when comparing the processing and correct categorization of each non-human primate species’ vocalizations to human voices using conjunction analyses, both subparts being crucially involved in this process (Dricu et al. 2017; Gruber et al. 2020). For all our hypotheses, we included the mean and standard deviation of vocalization fundamental frequency and energy as covariates (four covariates; random factors) to control for acoustic variability between species.

## Material and methods

### Experimental procedure and paradigm

**Fig. 1:**
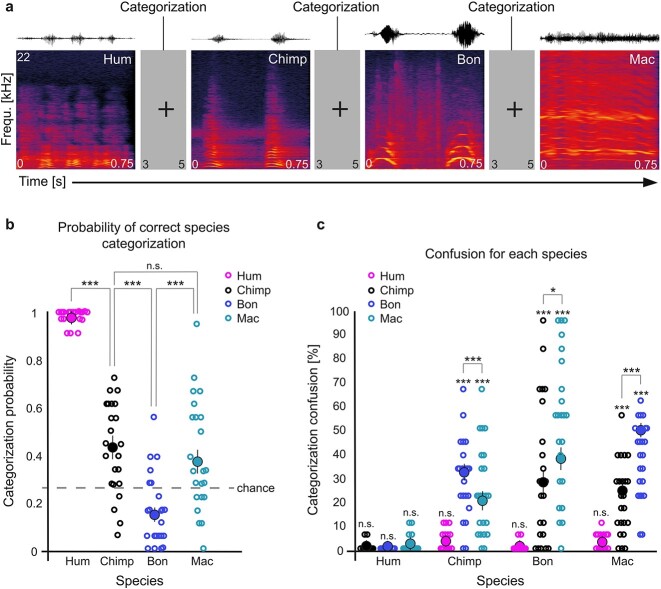
Experimental procedure and behavioral results of the probability of correct species categorization and confusions. a) Example of four consecutive experimental task trials (inter-trial interval of 3–5 s) for which the participants had to categorize the species’ stimulus (750 ms duration) for each trial (pseudo-randomized species order) and spectrogram example for each species. b) Probability of the correct categorization of each species (*y* axis) modeled across all participants, trials and species (i.e. human, chimpanzee, bonobo, macaque); each circle represents a participant’s averaged value per species, full circles represent the general average per species across participants ± the standard error of the mean, chance level is 25% (gray dotted line). c) Percentage of categorization confusion (*y* axis) specific to each species (*x* axis); each circle represents a participant’s averaged value, full circles represent the general average per species across participants ± the standard error of the mean. Hum: human; Chimp: chimpanzee; Bon: bonobo; Mac: macaque. ^***^p < 0.001, ^*^p < 0.05, n.s.: non-significant.

Participants laid comfortably in a 3T scanner and heard a total of seventy-two stimuli in pseudo-randomized order through MRI-compatible in-ear earphones (Sensimetrics Corporation, Gloucester, MA, USA) at a sound pressure level of 70 dB with the added sound attenuation of soft, 5 cm thick foam pads on each ear above the earphones. Participants were instructed to identify the species that expressed the vocalizations using a response box (Current Designs, Inc., Philadelphia, PA, USA). For instance, the instructions could be “Human – press 1, Chimpanzee – press 2, Bonobo – press 3 or Macaque – press 4”. There were four different mappings of the keys that were randomly assigned across participants. In a 3–5 s interval (jittering of 400 ms) after each stimulus, participants were asked to categorize the species. If the participant did not respond during this interval—namely a missed trial, the next stimulus followed automatically and the value of the response was “0” while “1” coded a correct response, “2” an incorrect response (Fig. 1a).

### Stimuli

Seventy-two vocalizations of four primate species (human, chimpanzee, bonobo and rhesus macaque) were used in this study. The eighteen human voices were expressed by two male and two female actors, obtained from a non-verbal (affective vocal burst) validated set of Belin and collaborators (Belin, Fillion-Bilodeau, et al. 2008b). Non-linguistic vocalizations were selected as human voice stimuli due to their acoustic and perceptual proximity with the other mammalian calls, non-human primates included (Anikin et al. 2018). The eighteen selected chimpanzee, bonobo and rhesus macaque vocalizations contained single calls or two call sequences produced by 6 to 8 different individuals in their natural environment. For each non-human primate species, the stimuli were either produced in an affiliative context or produced in an agonistic context (threating or distressful). All vocal stimuli were standardized to 750 milliseconds using PRAAT (www.praat.org) but were not normalized in order to preserve the “naturality” of the sounds (Ferdenzi et al. 2013). The impact of acoustic features on the evaluation of these stimuli were the focus of another article in which the reader can find all relevant information about this aspect, especially concerning the acoustic distance between these primate species (Debracque et al. 2023).

### Participants

Twenty-five right-handed, healthy, either native or highly proficient French-speaking participants took part in the study. One participant was excluded because he had no correct response at all and may have fallen asleep, while another participant was excluded due to incomplete scanning and technical issues, leaving us with twenty-three participants (10 female, 13 male, mean age 24.65 years, SD 3.66). All participants were naive to the experimental design and study, had normal or corrected-to-normal vision, normal hearing and no history of psychiatric or neurologic incidents and certified they did not encounter primates on a regular basis. Participants gave written informed consent for their participation in accordance with ethical and data security guidelines of the University of Geneva. The study was approved by the Ethics Cantonal Commission for Research of the Canton of Geneva, Switzerland (CCER) and was conducted according to the Declaration of Helsinki.

### Statistical analysis

Behavioral data were analyzed using R studio software (Team R 2020). In order to test our hypotheses regarding the impact of phylogenetic distance on species categorization, we performed mixed-effects models analyses of the “lme4” package (Bates et al. 2015) on accuracy and confusion (one accuracy column: “1”, correct response; “0”, incorrect response; four confusion columns: “1”, confusion with the species, “0” no confusion with the species, for each of the four species column) and reaction times data (for correct, incorrect trials and specific confusion for each species) with Species (human, chimpanzee, bonobo, macaque) and Affective context (affiliative and agonistic) as interacting fixed factors, four covariates (mean energy of each species, mean fundamental frequency of each species, energy standard deviation of each species, fundamental frequency standard deviation of each species) and the participants’ and the stimuli identity as random factors (random intercept and random slope, respectively). The inclusion of the sex and age of the participants for which we had no specific hypothesis in data modeling did not yield to a significantly better model neither for accuracy (χ^2^(2) = 0.81, p = 0.66) nor for reaction times data (χ^2^(2) = 0.01, p = 1) and these random effects were therefore discarded from the analyses. The same model was used to analyze accuracy, confusion and reaction times data, except that a logistic regression (generalized linear mixed-effects models, “glmer”) was used for accuracy and a linear regression (linear mixed-effects models, “lmer”) for specific pairs of species confusion (four models in total) and for reaction times. Then, we used specific contrasts computed using the “phia” package (De Rosario-Martinez et al. 2015) to compare between-species categorization, testing our hypothesis of an impact of phylogenetic proximity on species categorization. Contrasts therefore included human vs. non-human primate planned comparisons: [human > chimpanzee, bonobo, macaque vocalizations], [human > chimpanzee vocalizations], [human > bonobo vocalizations], [human > macaque vocalizations]; non-human primate vs. human comparisons: [chimpanzee > human vocalizations], [bonobo > human vocalizations], [macaque > human vocalizations]. These inverse contrasts actually yield to a sign-reversal of the difference estimate of the original contrast for accuracy data. For confusion data, contrasts were computed to uncover any difference between one human or non-human primate species compared to the remaining species: [Confusion for human vocalizations: chimpanzee response ≠ bonobo response ≠ macaque response], [Confusion for chimpanzee vocalizations: human response ≠ bonobo response ≠ macaque response], [Confusion for bonobo vocalizations: human response ≠ chimpanzee response ≠ macaque response], [Confusion for macaque vocalizations: human response ≠ chimpanzee response ≠ bonobo response]. All contrasts were corrected for multiple comparisons using a Bonferroni adjustment method implemented in the “phia” package (adjustment = “Bonf”) and *p*-value is accordingly reported as “*p_Bonf_*.” Reaction times data were analyzed using the same contrasts and are reported in Supplementary Material.

### Image acquisition

Structural and functional brain imaging data were acquired by using a 3T scanner Siemens Trio, Erlangen, Germany with a 32-channel coil. A 3D GR\IR magnetization-prepared rapid acquisition gradient echo sequence was used to acquire high-resolution (0.35 × 0.35 × 0.7 mm^3^) T1-weighted structural images (TR = 2,400 ms, TE = 2.29 ms). Functional images were acquired by using fast fMRI, with a multislice echo planar imaging sequence 79 transversal slices in descending order, voxel size = 3 × 3 × 3.9 mm, slice thickness 3 mm, TR = 650 ms, TE = 30 ms, field of view = 205 × 205 mm2, 64 × 64 matrix, flip angle = 50 degrees, bandwidth 1,562 Hz/Px.

### Wholebrain image analysis

Functional images were analyzed with Statistical Parametric Mapping software (SPM12, Wellcome Trust Centre for Neuroimaging, London, UK). Preprocessing steps included realignment to the first volume of the time series, slice timing, normalization into the Montreal Neurological Institute (MNI) space (Collins et al. 1994) using the DARTEL toolbox (Ashburner 2007) and spatial smoothing with an isotropic Gaussian filter of 8 mm full width at half maximum. To remove low-frequency components, we used a high-pass filter with a cut-off frequency of 128 s. Three general linear models were used to compute first-level statistics.

#### Model 1: Processing of all species, including all trials

In this model, each event was modeled by using a boxcar function and was convolved with the hemodynamic response function, time-locked to the onset of each stimulus. Separate regressors were created for all trials of each species (Species factor: human, chimpanzee, bonobo, macaque) and production context (Affective context factor: affiliative, agonistic) and four covariates each (mean energy of each species, mean fundamental frequency of each species, energy standard deviation of each species, fundamental frequency standard deviation of each species) for a total of 47 regressors. The means and standard deviations of the fundamental frequency and the energy of the vocalizations were extracted using the extended Geneva Acoustic parameters set, which is defined as the optimal acoustic indicators related to human voice analysis (GeMAPS (Eyben et al. 2016)). This set of acoustical parameters was selected based on i) their potential to index affective physiological changes in voice production, ii) their proven value in former studies as well as their automatic extractability, and iii) their theoretical significance.

Six motion parameters were included as regressors of no interest to account for movement in the data and our design matrix therefore included a total of 66 columns plus the constant term. The species regressors were used to compute simple contrasts for each participant, leading to separate main effects of human, chimpanzee, bonobo and macaque vocalizations for each production context (12 simple contrasts). Covariates were set to zero in order to model them as no-interest regressors. These simple contrasts were then taken to a flexible factorial second-level analysis in which there were three factors: the Participants factor (data independence set to “yes”, variance set to “unequal”), the Species factor and the Affective context factor (for both factors: data independence set to “no”, variance set to “unequal”). For this first model, the main effect of the Species factor was computed in the flexible factorial modeling while the Affective context factor was modeled but excluded from the results because we did not have any hypothesis regarding this factor and because each context modality contained only 6 trials per species.

#### Model 2: Model-based categorization probability of each species, including all trials

In this model, the probability of correctly categorizing each species in each production context was modeled using the “fitted” function of the “stats” package (Team RC 2013) to obtain the fitted probability values from the behavioral accuracy data described in the behavioral data analysis section. This modeling was performed by using each trial (N = 72) of each fMRI session (N = 1) of each participant (N = 23) with a binary “1” and “0” coding for the correct and incorrect species categorization, respectively, as dependent variable. As mentioned above, the model included fixed effects, namely the interaction between the Species factor (human, chimpanzee, bonobo, macaque) and the Affective context factor (affiliative, agonistic) and four covariates of interest (in this order: mean energy of each species, mean fundamental frequency of each species, energy standard deviation of each species, fundamental frequency standard deviation of each species). Random effects included the participants’ and the stimuli identity (random intercept and random slope, respectively). The sample-specific matrix (participants per trials = 23*72 = 1,656 lines) was then cut to the corresponding participant-specific matrix (72 lines). Model 1 therefore included two 72-lines regressors, namely the onsets of each trial in its order of appearance—modeled by using a boxcar function and convolved with the hemodynamic response function time-locked to the onset of each stimulus—and the corresponding modeled probability of correctly categorizing each species as a covariate of interest. Six motion parameters were included as regressors of no interest to account for movement in the data and our design matrix therefore included a total of 8 columns plus the constant term. The regressor of interest (modeled probability of correctly categorizing each species) was used to compute a simple contrast for each participant, leading to separate main effect of the probability of correctly categorizing each species. This simple contrast was then taken to a one-sample t-test second-level analysis in which variance was set to “unequal”.

#### Model 3: Correct categorization of each species, including only correct trials

This model is less complete than model 2, but helps clarify the message concerning phylogeny in our results. Separate regressors were created for each correctly categorized species (Correct Species factor: human hits, chimpanzee hits, bonobo hits, macaque hits vocalizations) for each production context (Affective context factor: affiliative, agonistic) and four covariates each (mean energy of each species, mean fundamental frequency of each species, energy standard deviation of each species, fundamental frequency standard deviation of each species) as well as a regressor containing a concatenation of categorization errors across species for a total of 44 regressors. Regressors were modeled by using a boxcar function and convolved with the hemodynamic response function, time-locked to the onset of each stimulus. Six motion parameters were included as regressors of no interest to account for movement in the data and our design matrix therefore included a total of 51 columns plus the constant term. The regressors of interest (Correct Species factor) were used to compute 8 simple contrasts for each participant, leading to separate main effects of human hits, chimpanzee hits, bonobo hits and macaque hits vocalizations for each production context. Covariates were set to zero in order to model them as no-interest regressors. These simple contrasts were then taken to a flexible factorial second-level analysis in which there were three factors: the Participants factor (independence set to “yes”, variance set to “unequal”), the Correct Species factor and the Affective context factor (for both factors: independence set to “no”, variance set to “unequal”). For this third model, the main effect of the Correct Species factor was computed in the flexible factorial modeling while the Affective context factor was modeled but excluded from the results because we did not have any hypothesis regarding this factor and because each modality contained only 6 trials per species.

**Fig. 2:**
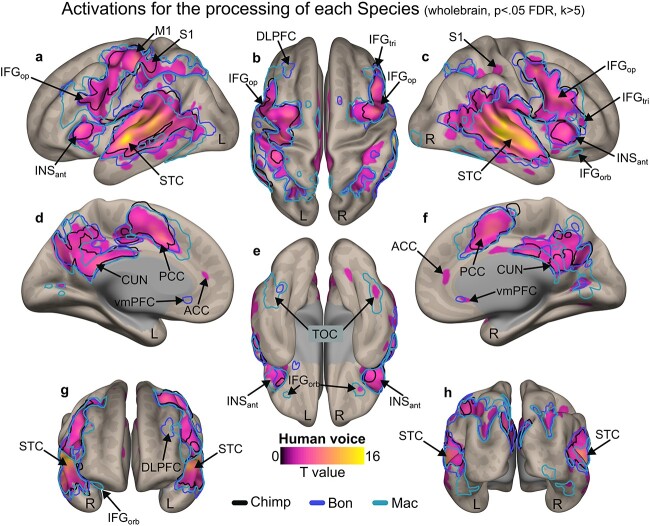
Wholebrain results of the processing of the species factor. Sagittal a and c), medial d and f), anterior g), posterior h), superior b) and inferior e) view of enhanced wholebrain activations for the processing of each species independently (main effect of species factor). For this model, trial-level covariates of no interest included the mean and standard deviation of the vocalization energy and fundamental frequency. The color bar illustrates t-value statistics. All activations thresholded at a voxelwise p < 0.05 FDR, k > 5 voxels. STC: superior temporal cortex—including the anterior, mid and posterior superior temporal gyrus and sulcus; M1: primary motor cortex; S1: primary somatosensory cortex; INS_ant_: anterior insula; DLPFC: dorsolateral prefrontal cortex; vmPFC: ventromedial prefrontal cortex; CUN: cuneus; PCC: posterior cingulate cortex; ACC: anterior cingulate cortex; TOC: temporo-occipital cortex; IFG: inferior frontal gyrus; tri: *pars triangularis*; op: *pars opercularis*; orb: *pars orbitalis*.

**Fig. 3:**
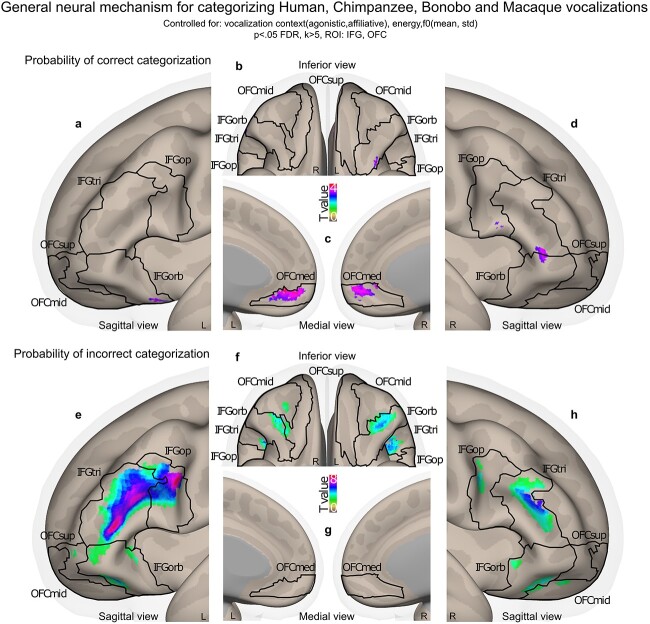
Model-based neural correlates and anti-correlates of the probability of correctly categorizing each species’ vocalizations in the IFG and OFC. a–d) Trial-by-trial correlates of the probability of correct species categorization. e–h) Trial-by-trial anti-correlates of the probability of correct species categorization. Data of this analysis were computed by using a modeling of species categorization accuracy across all participants, species (species factor) and trials including the affective context factor and trial-level covariates of no interest (mean and standard deviation of the vocalization energy and fundamental frequency). Subsequently, the fitted probability values of this model were used as a parametric modulator of interest for each trial’s onset, further taken as main regressor in second-level analyses. Color bars illustrate t-value statistics. All activations thresholded at a voxelwise p < 0.05 FDR, k > 5 voxels, masked by IFG and OFC regions of interest (k = 9,635 voxels in total). IFG: inferior frontal gyrus; tri: pars triangularis; op: pars opercularis; orb: pars orbitalis; OFC: orbitofrontal cortex; med: medial; mid: middle; sup: superior.

**Fig. 4:**
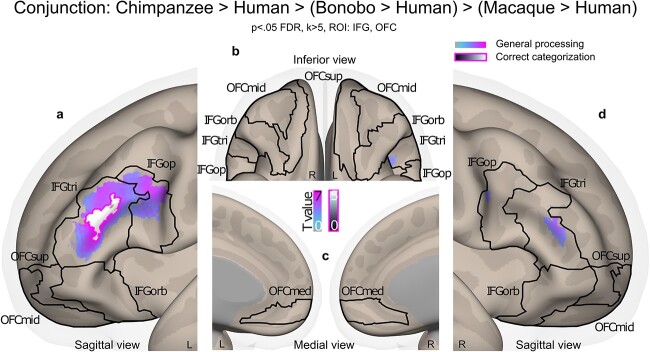
Conjunction results of the processing and correct categorization of non-human primates compared to human vocalizations in the IFG and OFC. a–d, Conjunction analysis for the processing (all trials of each species) and correct species categorization (correct trials only) of the contrasts [(chimpanzee > human) > (bonobo > human) > (macaque > human) vocalizations]. No above-threshold voxels were found for [bonobo > human] or [macaque > human] contrasts driving the conjunction analyses. For both models (species processing; correct species categorization), trial-level covariates of no interest included the mean and standard deviation of the vocalization energy and fundamental frequency. Colorbars illustrate t-value statistics. All activations thresholded at a voxelwise p < .05 FDR, k > 5 voxels, masked by IFG and OFC regions of interest (k = 9635 voxels in total). IFG: inferior frontal gyrus; tri: pars triangularis; op: pars opercularis; orb: pars orbitalis; OFC: orbitofrontal cortex; med: medial; mid: middle; sup: superior.

For model 1, data were not masked for Fig. 2 and are therefore “wholebrain”. For all other displays and illustrations and to be consistent in our analyses, neuroimaging activations were masked by a binary image including all subparts of the IFG (*pars opercularis*, *triangularis* and *orbitalis*) and OFC (superior, middle and medial) for a total of twelve subregions (9,635 voxels) and thresholded in SPM12 by using a voxel-wise false discovery rate (FDR) correction at p < 0.05 and an arbitrary cluster extent of k > 5 voxels to remove extremely small clusters of activity or single-voxel activations. Region masks were extracted using the “automated anatomical labelling” (“aal”) atlas (Tzourio-Mazoyer et al. 2002) implemented in the “WFU_Pickatlas” toolbox (https://www.nitrc.org/projects/wfu_pickatlas/). Contrast activations were rendered on semi-inflated brains from the CONN toolbox (Whitfield-Gabrieli and Nieto-Castanon 2012) with black outlines delineating each of our IFG-OFC regions of interest in Figs 3 and 4 as well as in Supplementary Figs. S4–S7.

## Results

The present study aimed at assessing the ability of humans to accurately classify human and more importantly other primate calls, in the present case chimpanzee, bonobo, and macaque vocalizations (Fig. 1a) and how frontal brains areas may underlie such successful stimuli categorization. Behavioral analyses were performed using a Species factor (human, chimpanzee, bonobo, macaque) interacting with the Affective context factor for each vocalization (affiliative, agonistic “threat”, agonistic “distress”) in a generalized linear mixed-effects modeling of the accuracy data (“1”, correct response; “0”, incorrect response) and a linear mixed-effects modeling of the confusion data. This statistical procedure was used to better assess the probability of correctly categorizing each species’ vocalizations as well as the pattern of confusion between each species, respectively. Following similar reasoning, we used the trial-level probability of correct species categorization in model-based fMRI methodology. We tested the specific frontal mechanism underlying the combined processing and accurate classification of each non-human primate species vocalizations compared to human voices using conjunction analyses. Both analyses were conducted in our regions of interest (ROI), namely in the bilateral IFG (*pars opercularis*, *triangularis*, *orbitalis*) and OFC (superior, middle, medial) using masked second-level analyses with voxelwise corrected statistics (p < 0.05 False Discovery Rate correction “FDR”, k > 5 voxels). Selected low-level acoustics (mean and standard deviation of stimulus energy and fundamental frequency) were used as covariates of no interest for behavioral and neuroimaging analyses to better control our data for low-level vocal fluctuations. Statistical modeling power is reported for fixed effects (“R^2^c”) and for both fixed and random effects taken together (“R^2^m”).

### Behavioral results

#### Accuracy data

As predicted, the generalized linear mixed-effects on accuracy revealed a significant effect of Species factor (χ^2^(3) = 222.78, p < 0.0001, R^2^c = 56.38% and R^2^m = 61.10%). All complementary results of this model can be found in Supplementary Table S1. We then tested the contrasts of interest according to our behavioral hypothesis namely the impact of phylogenetic proximity on accurate species categorization: our results highlighted a higher probability of correctly categorizing human compared to the vocalizations of each non-human primate (χ^2^(1) = 128.96, *p_Bonf_* < 0.0001; human > chimpanzee: χ^2^(1) = 95.89, *p_Bonf_* < 0.0001; human > bonobo: χ^2^(1) = 199.51, *p_Bonf_* < 0.0001; human > macaque: χ^2^(1) = 79.40, *p_Bonf_* < 0.0001). We additionally compared each non-human primate species with each other and found a higher probability of correct categorization for chimpanzee compared to bonobo (χ^2^(1) = 52.64, *p_Bonf_* < 0.0001), and macaque compared to bonobo vocalizations (χ^2^(1) = 18.99, *p_Bonf_* < 0.0001). No difference was observed when comparing the probability of correct species categorization between chimpanzee and macaque vocalizations (χ^2^(1) = 1.84, *p_Bonf_* = 0.17, Fig. 1b).

Confusion data analyses were computed by considering species miscategorization, namely one species’ vocalization being categorized as belonging to another species (Fig. 1c). The confusion for human voices was not significant (χ^2^(3) = 1.65, p = 0.65), meaning that these were not significantly miscategorized with any other species. Confusion concerning chimpanzee vocalizations showed a significant main effect of Species (χ^2^(3) = 265.79, p < 0.0001; R^2^c = 16.41% and R^2^m = 24.64%), showing that these were mostly miscategorized as bonobo vocalizations (χ^2^(1) = 149.52, *p_Bonf_* < 0.0001), and then as macaque (χ^2^(1) = 47.11, *p_Bonf_* < 0.0001) vocalizations (for chimpanzee confusions, bonobo > macaque: χ^2^(1) = 15.34, *p_Bonf_* < 0.0001), but not as human voices (χ^2^(1) = 0.21, *p_Bonf_* > 0.99). Confusion for bonobo vocalizations showed a significant main effect of the Species factor (χ^2^(3) = 107.96, p < 0.0001; R^2^c = 24.50% and R^2^m = 28.95%) and specific confusion was the highest with macaque (χ^2^(1) = 114.70, *p_Bonf_* < 0.0001) and then chimpanzee (χ^2^(1) = 111.28, *p_Bonf_* < 0.0001) vocalizations (for bonobo confusions, macaque > chimpanzee: χ^2^(1) = 5.33, *p_Bonf_* < 0.05) but no significant confusion with human voices was observed (χ^2^(1) = 0.06, *p_Bonf_* > 0.99). Confusion for macaque vocalizations showed a main effect of the Species factor (χ^2^(3) = 469.68, p < 0.0001; R^2^c = 29.01% and R^2^m = 30.65%) with highest confusion with bonobo (χ^2^(1) = 565.04, *p_Bonf_* < 0.0001) followed by chimpanzee (χ^2^(1) = 145.96, *p_Bonf_* < 0.0001) vocalizations (for macaque confusions, bonobo > chimpanzee: χ^2^(1) = 87.34, *p_Bonf_* < 0.0001). No confusion with the human voice reached significance (χ^2^(1) = 0.04, *p_Bonf_* > 0.99). All other specific confusion effects can be found in Supplementary Table S2.

### Neuroimaging results: Wholebrain and using IFG and OFC as regions of interest

We first display the general, wholebrain results of the main effect of the Species factor, including the activations of the processing of each species separately (model 1). We then used a model-based approach in order to characterize the general frontal mechanism underlying the accurate categorization of primate vocalizations by human participants (see model 2 in the methods section). To do so, we modeled all trials with the sample-specific fitted probability of a correct vs. incorrect species categorization as a covariate of interest for each trial’s onset. This covariate was then taken as the main regressor of interest to masked group-level analyses to reveal frontal patterns of activity related to the general human neural mechanism of classifying human, chimpanzee, bonobo, and macaque vocalizations. These fitted probability values were obtained across participants and species by predicting accuracy data at the trial level, therefore including all trials, correct and incorrect, in a logistic regression analysis. Fixed effects included the Species and Affective context factors and covariates of no interest (mean and standard deviation of vocalization energy and fundamental frequency) with random effects characterizing the identity of each participant (random intercept) and each stimulus (random slope). We finally present a conjunction analysis focusing on the direct multiple comparison between the processing and correct evaluation of each primate species’ calls as opposed to the human voice, following phylogeny.

Wholebrain activations revealed expected large clusters of activations in the superior temporal cortex (STC), inferior frontal and insular cortex (Fig. 2a–c) as well as in the ventromedial prefrontal cortex—including the OFC, Fig. 2f—and in primary motor and somatosensory brain areas (Fig. 2a and c). While there was a massive overlap in the activations for the processing of each species, we should highlight that the processing of macaque calls recruited left inferior frontal regions more than any other primate species in our data (teal outline, Fig. 2a and g), and that chimpanzee calls showed a recruitment of brain areas very similar to those of human voice processing (Fig. 2a–d and f–h, black outline). Please refer to Supplementary Figs S1–S3 for the full activation maps—including color bars—of each non-human primate species and Supplementary Table S3 for region coordinates of Fig. 2.

We then used computational modeling of our neuroimaging data to tackle our hypothesis on the general neural mechanisms underlying the probability of a correct and incorrect species categorization of each species by our human participants. Activity correlating with the probability of correctly categorizing each species’ vocalizations was revealed in the right IFG_tri_ (Fig. 3b) and bilateral IFG_orb_ (Fig. 3ab and d) as well as in the bilateral OFC_med_ (Fig. 3c). Anti-correlated activity was revealed in a large left IFG_tri_, IFG_op_ cluster (Fig. 3e and f) and to a lesser extent in the left IFG_orb_ (Fig. 3e), right IFG_op_ and IFG_orb_ (Fig. 3f and h) and in the right OFC_mid_ (Fig. 3b) and left OFC_sup_ (Fig. 3e). See Supplementary Table S4 for cluster coordinates.

We finally computed two conjunction analyses with the vocalizations of each non-human primate species compared to human voice categorization. In these analyses, enhanced activations for each non-human primate species was tested while driving the conjunction but only the [(chimpanzee > human) > (bonobo > human) > (macaque > human) vocalizations] contrast showed significant results (see model 2 in the methods section). This conjunction revealed left-lateralized IFG_tri_ and IFG_op_ as well as right IFG_tri_ activity for the processing of the vocalizations (i.e. general processing displayed as white-to-pink activations, Fig. 4a, b and d) while enhanced activity for correctly categorized species (see model 3 in the methods section) was only found in left IFG_tri_ (i.e. correct categorization displayed as black-to-white activations, Fig. 4a). No enhanced signal was observed in the OFC. Other combinations of non-human primate vs. human species conjunction contrasts did not yield to any above-threshold voxels. Mean and standard deviation of each vocalization’s energy and fundamental frequency were used as trial-level covariates of no interest in these statistical analyses as well. See Supplementary Table S5 for cluster coordinates.

The comparisons between the processing (all trials) or the correct categorization (correct trials only) of the vocalizations of each non-human species compared to those of humans, and of human voices compared to the vocalizations of each non-human primate species are reported in Supplementary Figs S4 and S5 respectively. Activations specific to the processing and correct categorization of each non-human primate species or between each non-human primate species are reported in Supplementary Figs S6 and S7, respectively.

## Discussion

The present study was designed to clarify the importance of primate vocalizations processing and classification by humans as a proxy for the more general topic of human communication. In fact, the processing of primate vocalizations by humans may boost our understanding of non-verbal communication in today’s *H. sapiens* and inform on the ties of our last common ancestor with other Hominids. To operationalize such a research objective, we investigated modern humans behavioral and frontal brain mechanisms underlying the vocal recognition of non-human primates’ vocalizations, considering their phylogenetic proximity to the human species. Our data highlighted the evolutionary implications of our species with the communication system of other primates—particularly other great apes—with both the recognition and classification processes of hominid vocalizations. This process involved the medial orbitofrontal and bilateral inferior frontal cortex, especially the *pars triangularis*.

Behavioral data emphasized the astonishing ability of human participants to categorize chimpanzee and macaque calls above chance level while it was not the case for bonobo vocalizations. Despite the close phylogenetic proximity of this species with *H. sapiens*, the peculiar acoustical structure of bonobo calls may have prevented modern humans from recognizing them, yielding to miscategorization. Indeed, confusion matrices showed that, despite their close phylogenetic relationship to chimpanzees, bonobo vocalizations were in fact often confused for those of macaques, or maybe randomly assigned to either category as a potential hesitation strategy. This peculiarity in the processing of bonobo vocalizations has also been reported in previous literature showing that human adults are highly sensitive to pitch information for discriminating the arousal of primate babies’ cries (Kelly et al. 2017). In fact, human participants rated bonobo infant calls as more distressful in comparison to those of humans or chimpanzees and after further acoustical analyses, the authors found that bonobo infants’ calls had higher pitched vocalizations compared to those of human and chimpanzee babies, both species sharing more similar acoustical features (Kelly et al. 2017). Such acoustical divergence of bonobos among non-human primates and especially the increased fundamental frequency of their calls may well have its origin in the shorter length of the larynx of this species (Grawunder et al. 2018). Interestingly in our study, chimpanzee calls were not better categorized by human participants than those of a more evolutionary distant species such as rhesus macaques. This was especially surprising considering that previous studies have mostly found a better recognition rate for chimpanzee vocalizations compared to the calls of other non-human primates (Linnankoski et al. 1994; Filippi et al. 2017). Hence, our behavioral analysis suggests that the capacity of modern humans to accurately identify non-human primate calls could rely more heavily on acoustic similarity rather than phylogenetic proximity alone (Debracque et al. 2023).

To assess the frontal brain mechanisms involved in the general processing of human voice and non-human primate vocalization recognition, we turned to computational modeling and more precisely to model-based fMRI analyses. These data pointed to a frontal network including the bilateral medial OFC for the correct recognition and categorization of human voices and non-human primate vocalizations. This first result suggests that the medial OFC has a central role in the accurate categorization of primate calls, extending previous fMRI results that showed an enhancement of activity in OFC regions—especially of the medial part—for the discrimination of emotions in human, chimpanzee, macaque, and cat vocalizations (Belin, Fecteau, et al. 2008a; Fritz et al. 2018). Other frontal brain areas underlying the probability of accurate species categorization included small clusters of the bilateral IFG_orb_ as well as the right IFG_op_ and IFG_tri_. Frontal brain areas underlying miscategorization and/or what could be labeled as “hesitation” were found in large portions of the bilateral IFG, especially in the IFG_tri_ which is coherent with existing literature (Moss et al. 2005). This result is also supported by our conjunction analyses, which allowed a more concise and focused multi-contrast approach to pinpoint the specific frontal brain mechanisms that may underlie the processing as well as accurate recognition and categorization of non-human primate vocalizations compared to the human voice. These analyses revealed large, similar bilateral IFG_tri_ clusters of activity when creating a hierarchy for processing chimpanzee, bonobo and macaque vocalizations each compared to the human voice—namely, contrasting (chimpanzee > human) > (bonobo > human) > (macaque > human) at the same time. Interestingly, this conjunction contrast also revealed a difference in the hierarchy between the accurate recognition of chimpanzee vocalizations and those of bonobos and macaques when each was compared to the human voice in a subpart of the left IFG_tri_. In the literature, the IFG_tri_ was repeatedly involved when human participants have to make a decision based on vocal signals, for instance when human participants discriminate or categorize human vocal emotion (Dricu et al. 2017; Gruber et al. 2020) or when they implicitly or explicitly process human emotional voices (Frühholz et al. 2012). Causal links between IFG_tri_ functioning and emotion recognition were also revealed in the literature (Keuken et al. 2011). Noteworthy is the fact that activity in the left IFG_tri_ also translates a level of uncertainty when decisional processes occur (Moss et al. 2005; Nastase et al. 2014; Toelch et al. 2014; Ceravolo et al. 2021). Combining our behavioral and neuroimaging results, it seems extremely plausible that decisional uncertainty was peaking when human participants were categorizing non-human primate vocalizations. We therefore think that according to our results and to the existing literature, activity in the IFG_tri_ might underlie both categorization uncertainty as revealed by anti-correlates of the probability of accurate species recognition in the model-based neuroimaging results, as well as decisional processes that lead to an accurate species recognition in a very concise region of the left IFG_tri_, as highlighted by our conjunction results. Therefore, OFC and IFG brain areas—especially IFG_tri_ activity—may be the necessary biological and functional “hardware” allowing *H. sapiens* to classify and integrate vocal communication, be it human voice or other primate vocalizations. Such ability may then have been co-opted for the treatment of the proto-language used by our human ancestors, although precisely how close this proto-language was from to today’s non-human primates vocalizations remains an open question, with a question mark regarding a potential interaction with between-species acoustical differences (Debracque et al. 2023). We contend that the common activity we evidenced in the OFC and IFG across stimuli suggests that such proto-language may have shared acoustic properties with non-human primate vocalizations.

Even though our results shed new light on the origins of human communication, our study suffered from several limitations. Such limitations include the absence of higher temporal resolution, the absence of other great apes as stimuli (for instance gorillas and/or orangutans) or of other more remote species and the absence of highly specific acoustic distance assessment. Also, the time-course of such frontal mechanisms underlying non-verbal communication in humans should be investigated.

To conclude, the present study revealed the existence of distinct mechanisms at play in the human frontal cortex for the integration and further categorization of non-human primate vocalizations. In fact, computational modeling of the fMRI data allowed a clear distinction between accurate categorization on the one hand and miscategorization processes on the other hand, as revealed by bilateral medial OFC and left IFG_tri_, respectively. Combined with our behavioral data, evidence converges toward a preserved ability of *H. sapiens* to recognize the calls of species close to his ancestors that were most likely very similar to those of today’s non-human primate vocalizations. Natural selection might also have led to specific anatomical adaptations in some non-human primates across time, and the shorter larynx of bonobos leading to the significantly increased fundamental frequency of their calls is a well-fitting example, especially in the context of the present study. Therefore, a combination of phylogenetic and acoustical proximity may be necessary for humans to recognize and successfully categorize the calls of non-human primates, including those of great apes. Overall, the present study substantially contributes to the understanding of primate calls’ recognition by modern humans as a potential proxy for more general communication mechanisms in *H. sapiens*.

## Supplementary Material

Ceravolo_SupplementaryData_tgad019

## Data Availability

All data and codes used in the present study are available at a public, non-profit repository (https://yareta.unige.ch/ and https://doi.org/10/gs3w6t and https://doi.org/10/gs3w6t).

## CRediT author statement

Leonardo Ceravolo (Conceptualization, Formal analysis, Methodology, Software, Validation, Writing—original draft, Writing—review & editing), Coralie Debracque (Conceptualization, Formal analysis, Validation, Writing—review & editing), Eva Pool (Formal analysis, Methodology, Writing—review & editing), Thibaud Gruber (Funding acquisition, Project administration, Resources, Supervision, Validation, Writing—review & editing), and Didier Grandjean (Conceptualization, Funding acquisition, Project administration, Resources, Supervision, Validation, Writing—review & editing)
