# Supplementary material for: Frontal mechanisms underlying primate calls recognition by humans
Source: Cereb Cortex Commun. 2023 Nov 2;4(4):tgad019. doi: 10.1093/texcom/tgad019 (PMC10661312; doi:10.1093/texcom/tgad019)
Supplement: Ceravolo_SupplementaryData_tgad019 [file ceravolo_supplementarydata_tgad019.pdf]

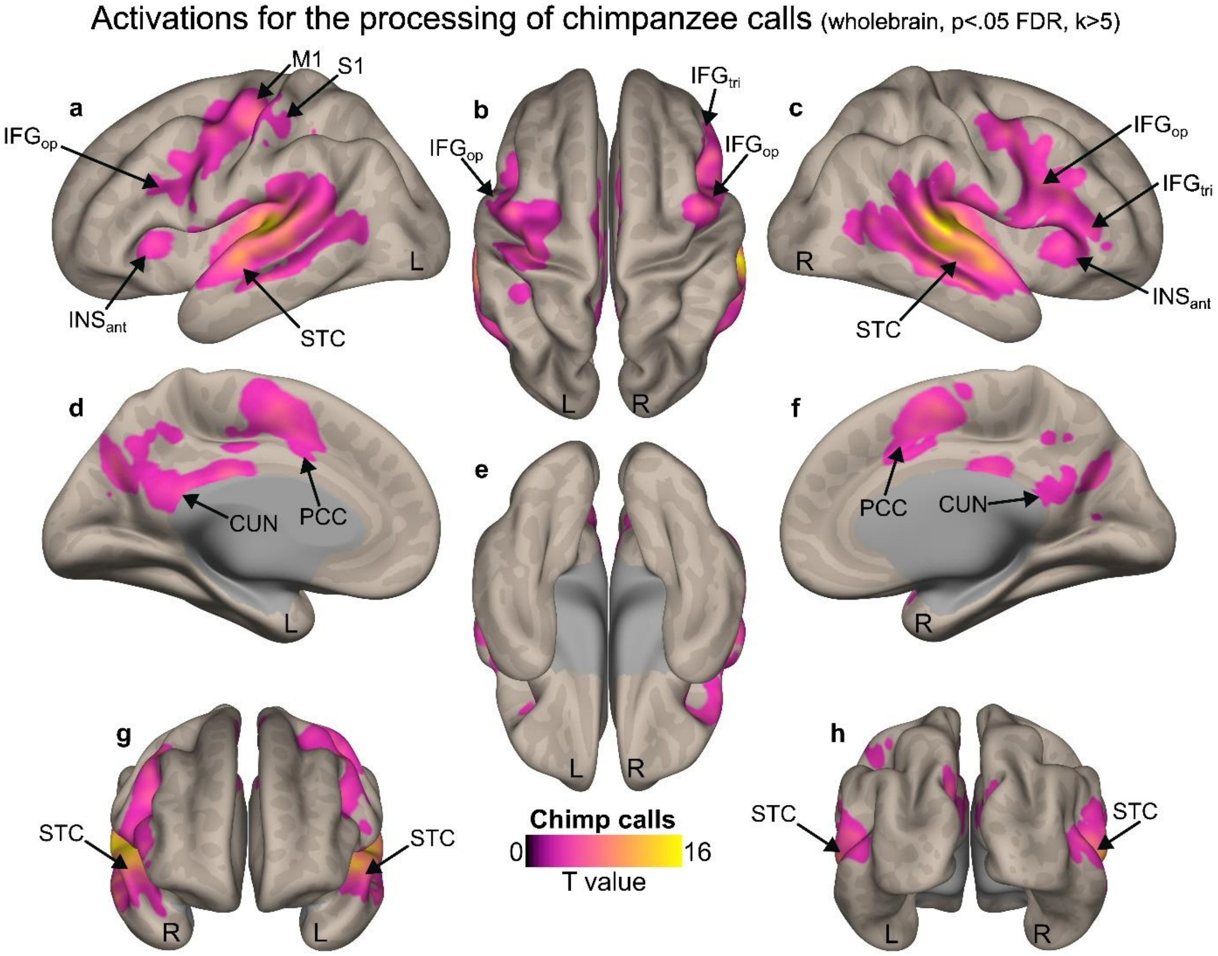

Fig.S1: Wholebrain results of the processing of the Species factor, chimpanzee calls. Sagittal (**ac**), medial (**df**), anterior (**g**), posterior (**h**), superior (**b**) and inferior (**e**) view of enhanced wholebrain activations for the processing of chimpanzee calls (main effect of Species factor). For this model, trial-level covariates of no interest included the mean and standard deviation of the vocalization energy and fundamental frequency. The colorbar illustrates t-value statistics. All activations thresholded at a voxelwise $p<.05$ FDR, k>5 voxels. STC: superior temporal cortex—including the superior temporal gyrus and sulcus; M1: primary motor cortex; S1: primary somatosensory cortex; INS_{ant}: anterior insula; CUN: cuneus; PCC: posterior cingulate cortex; IFG: inferior frontal gyrus; tri: pars triangularis; op: pars opercularis.

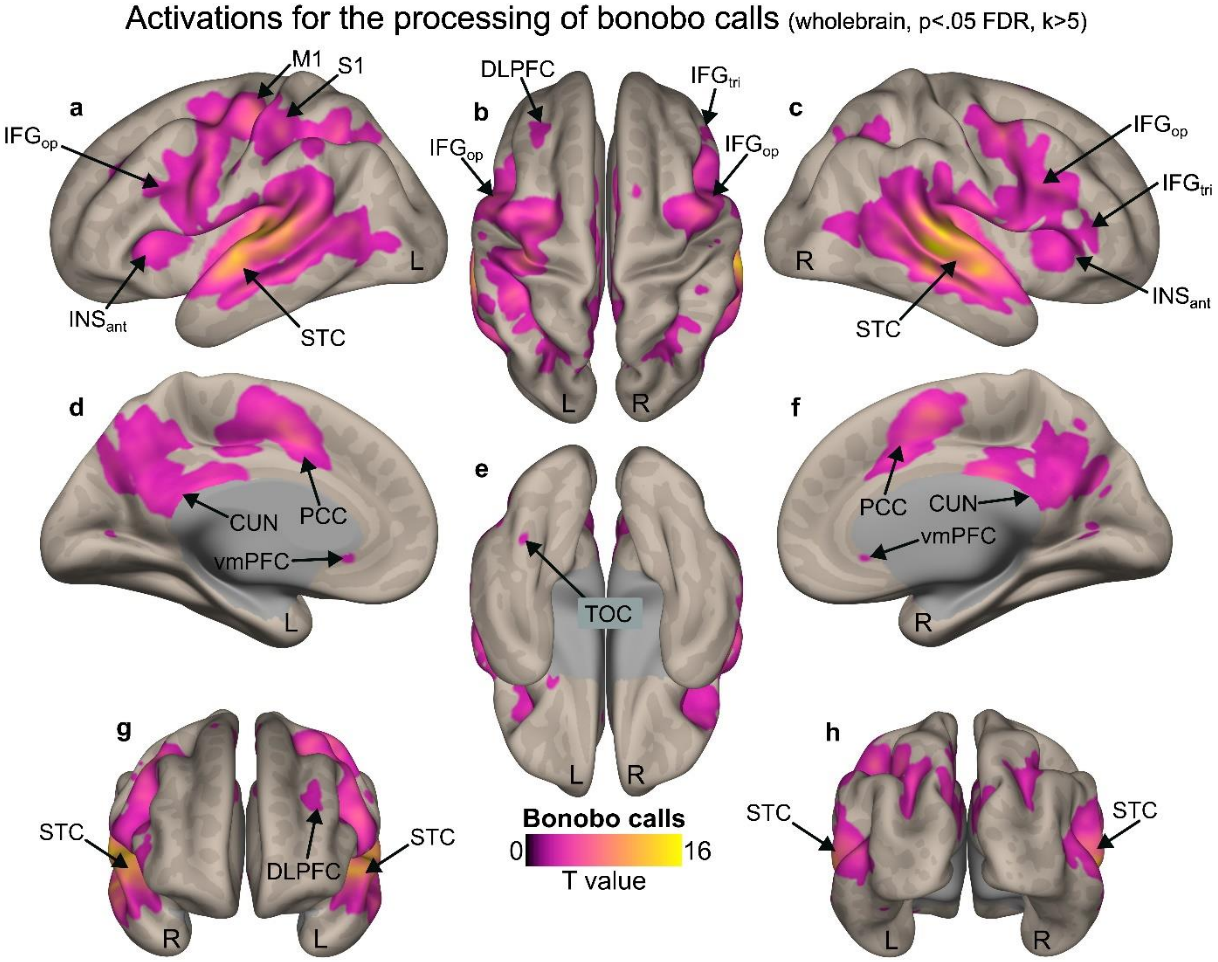

Fig.S2: Wholebrain results of the processing of the Species factor, bonobo calls. Sagittal (**ac**), medial (**df**), anterior (**g**), posterior (**h**), superior (**b**) and inferior (**e**) view of enhanced wholebrain activations for the processing of bonobo calls (main effect of Species factor). For this model, trial-level covariates of no interest included the mean and standard deviation of the vocalization energy and fundamental frequency. The colorbar illustrates t-value statistics. All activations thresholded at a voxelwise p<.05 FDR, k>5 voxels. STC: superior temporal cortex—including the superior temporal gyrus and sulcus; M1: primary motor cortex; S1: primary somatosensory cortex; INS_{ant}: anterior insula; DLPFC: dorsolateral prefrontal cortex; vmPFC: ventromedial prefrontal cortex; CUN: cuneus; PCC: posterior cingulate cortex; TOC: temporo-occipital cortex; IFG: inferior frontal gyrus; tri: pars triangularis; op: pars opercularis.

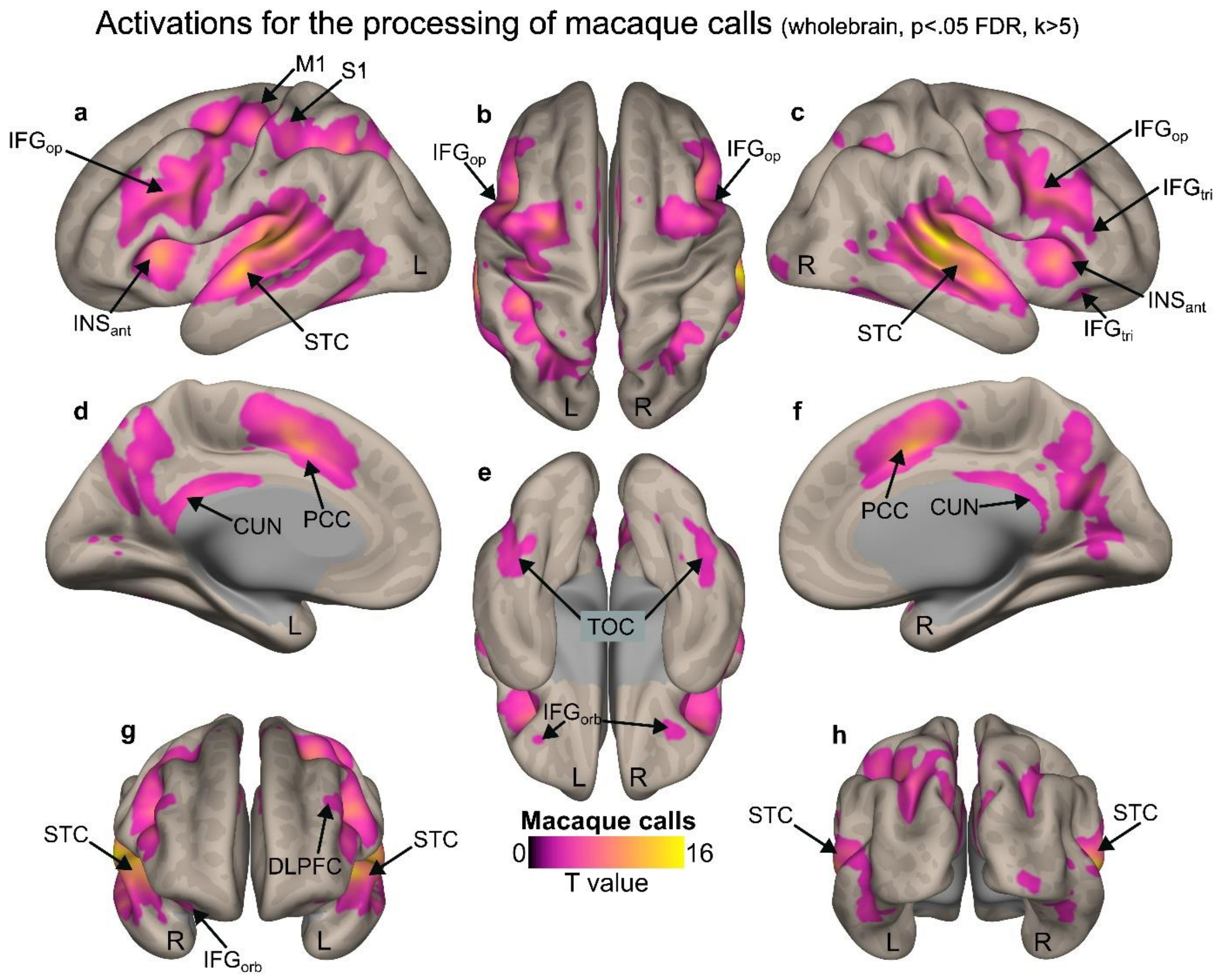

Fig.S3: Wholebrain results of the processing of the Species factor, macaque calls. Sagittal (**ac**), medial (**df**), anterior (**g**), posterior (**h**), superior (**b**) and inferior (**e**) view of enhanced wholebrain activations for the processing of macaque calls (main effect of Species factor). For this model, trial-level covariates of no interest included the mean and standard deviation of the vocalization energy and fundamental frequency. The colorbar illustrates t-value statistics. All activations thresholded at a voxelwise $p<.05$ FDR, k>5 voxels. STC: superior temporal cortex—including the superior temporal gyrus and sulcus; M1: primary motor cortex; S1: primary somatosensory cortex; INS_{ant}: anterior insula; DLPFC: dorsolateral prefrontal cortex; CUN: cuneus; PCC: posterior cingulate cortex; TOC: temporo-occipital cortex; IFG: inferior frontal gyrus; tri: pars triangularis; op: pars opercularis; orb: pars orbitalis.

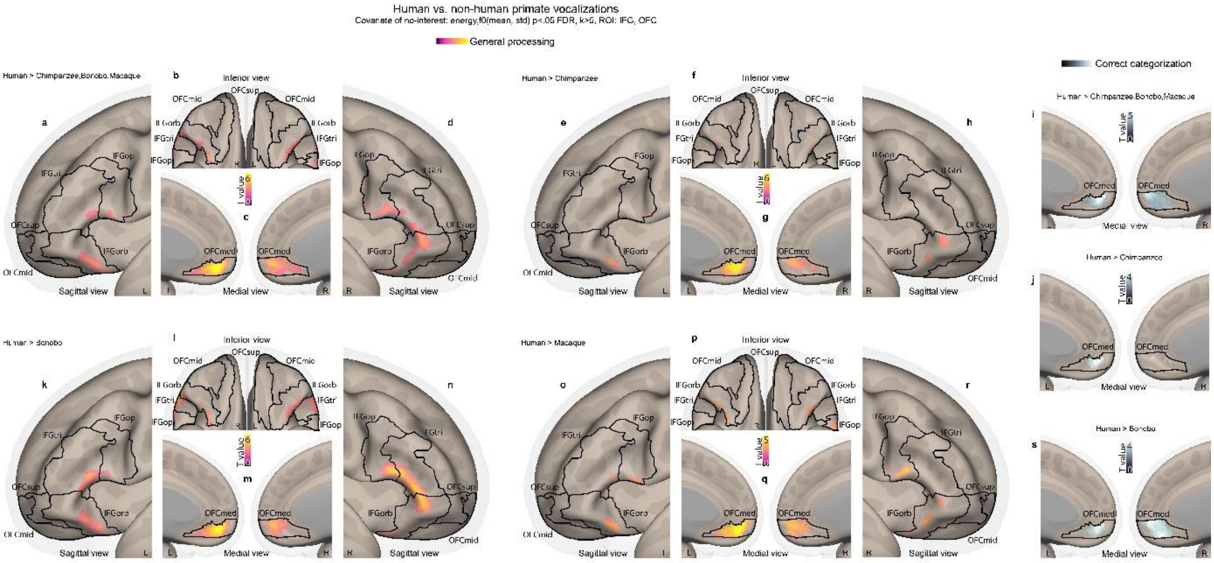

Fig.S4: Neural activations of the processing and correct categorization of human compared to non-human primates vocalizations in the IFG and OFC. Enhanced activations for the processing (all trials of each species) of [human > chimpanzee, bonobo, macaque vocalizations, **abcd**], [human > chimpanzee vocalizations, **efgh**], [human > bonobo vocalizations, **klmn**] and [human > macaque vocalizations, **opqr**]. Enhanced activations for the correct categorization (correct trials only) of [human > chimpanzee, bonobo, macaque vocalizations, **i**], [human > chimpanzee vocalizations, **j**] and [human > bonobo vocalizations, **s**]. No above-threshold voxels were found for correctly categorized [human > macaque vocalizations]. For both models (species processing; correct species categorization), trial-level covariates of no interest included the mean and standard deviation of the vocalization energy and fundamental frequency. Colorbars illustrate t-value statistics, with purple-to-yellow bars and activations used for contrasts of species processing while black-to-white was used for correct species categorization. All activations thresholded at a voxelwise p<.05 FDR, k>5 voxels, masked by IFG and OFC regions of interest (k=9635 voxels in total). IFG: inferior frontal gyrus; tri: pars triangularis; op: pars opercularis; orb: pars orbitalis; OFC: orbitofrontal cortex; med: medial; mid: middle; sup: superior.

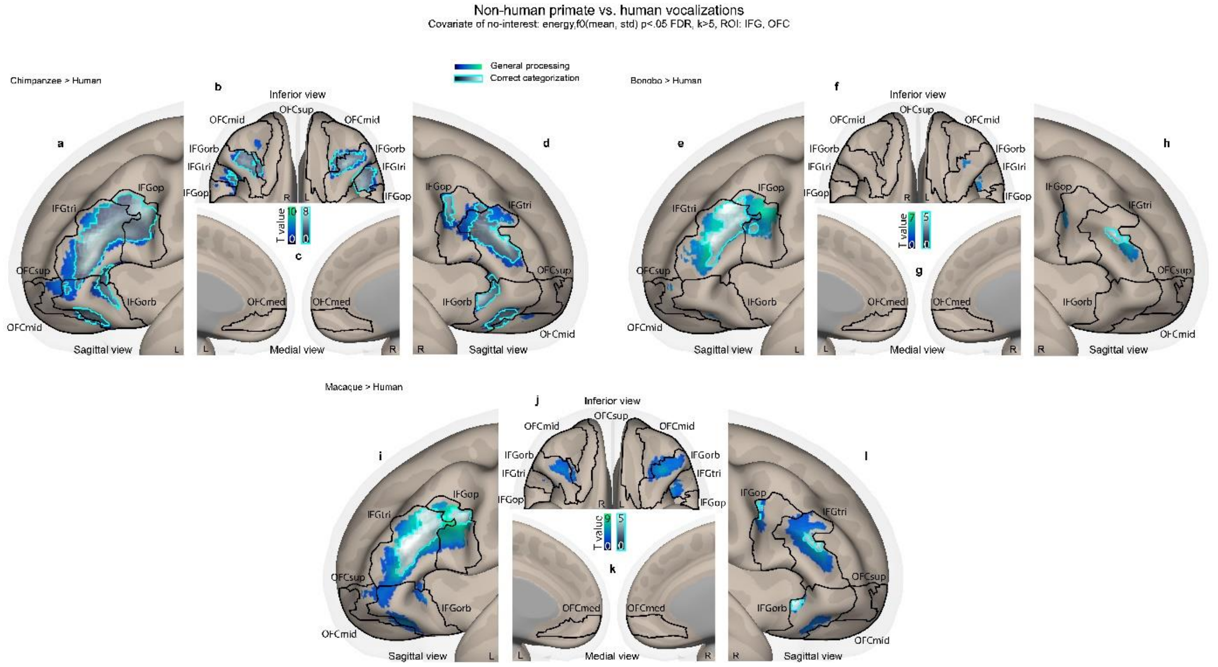

Fig.S5: Neural activations of the processing and correct categorization of non-human primates compared to human vocalizations in the IFG and OFC. Enhanced activations for the processing (blue-to-green activations, all trials of each species) and correct species categorization (black-to-white activations with teal outline, correct trials only) of [chimpanzee > human vocalizations, **abcd**], [bonobo > human vocalizations, **efgh**] and [macaque > human vocalizations, **ijkl**]. For both analyses (species processing; correct species categorization), trial-level covariates of no interest included the mean and standard deviation of the vocalization energy and fundamental frequency. Colorbars illustrate t-value statistics. All activations thresholded at a voxelwise *p*<.05 FDR, k>5 voxels, masked by IFG and OFC regions of interest (k=9635 voxels in total). IFG: inferior frontal gyrus; tri: pars triangularis; op: pars opercularis; orb: pars orbitalis; OFC: orbitofrontal cortex; med: medial; mid: middle; sup: superior.

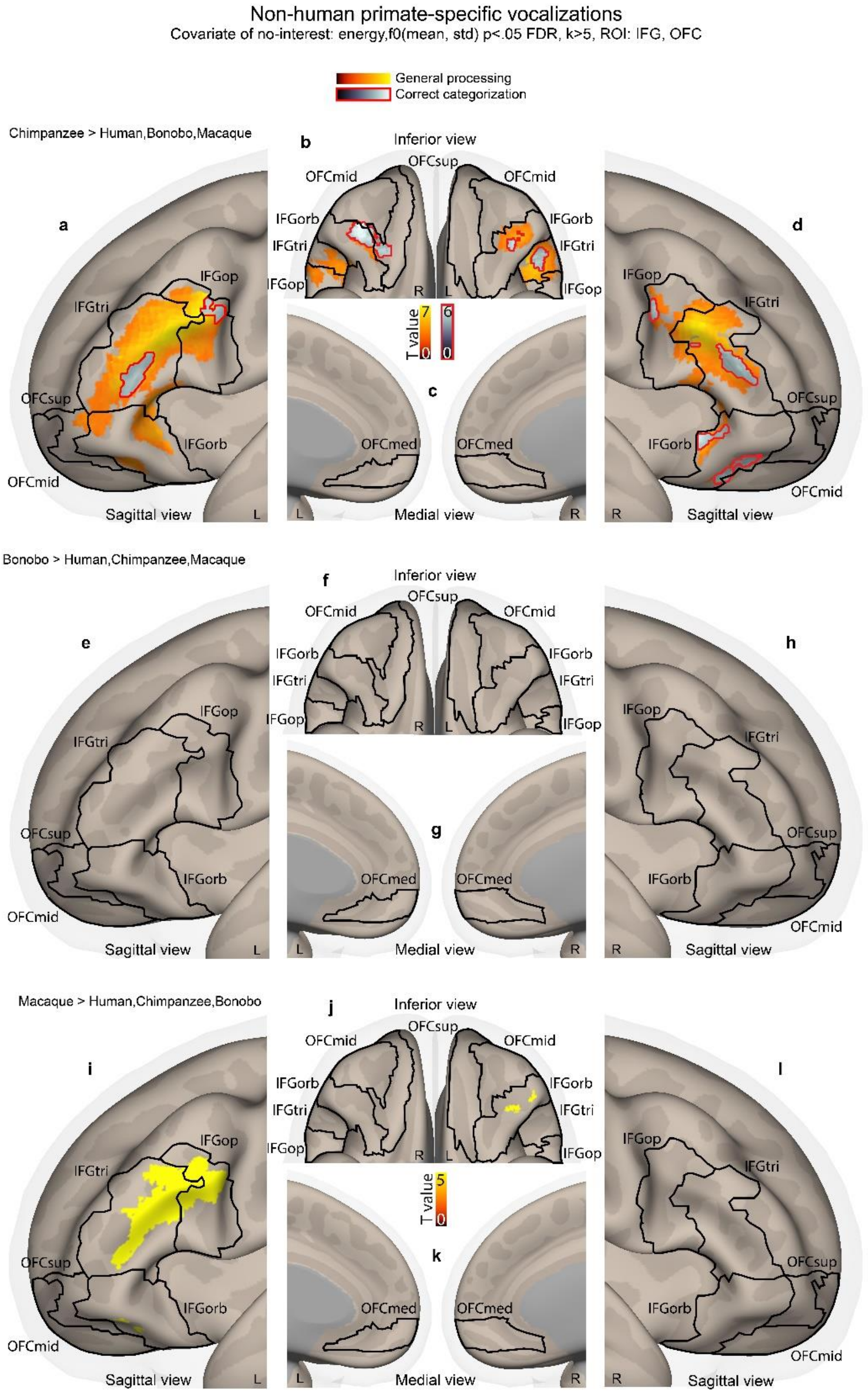

Fig.S6: Brain activations specific to each non-human primate species within the IFG and OFC. abcd: Processing and correct responses for Chimpanzee > Bonobo, Macaque, Human vocalizations. **efgh**: Bonobo > Chimpanzee, Macaque, Human vocalizations processing. **ijkl**: Macaque > Chimpanzee, Bonobo, Human vocalizations processing. For both models (species processing; correct species categorization), trial-level covariates of no interest included the mean and standard deviation of the vocalization energy and fundamental frequency. Colorbars illustrate t-value statistics, with red-to-yellow bars and activations used for contrasts of species processing while black-to-white with red outline was used for correct species categorization. All activations thresholded at a voxelwise p<.05 FDR, k>5 voxels, masked by IFG and OFC regions of interest (k=9635 voxels in total). IFG: inferior frontal gyrus; tri: *pars triangularis*; op: *pars opercularis*; orb: *pars orbitalis*; OFC: orbitofrontal cortex; med: medial; mid: middle; sup: superior.

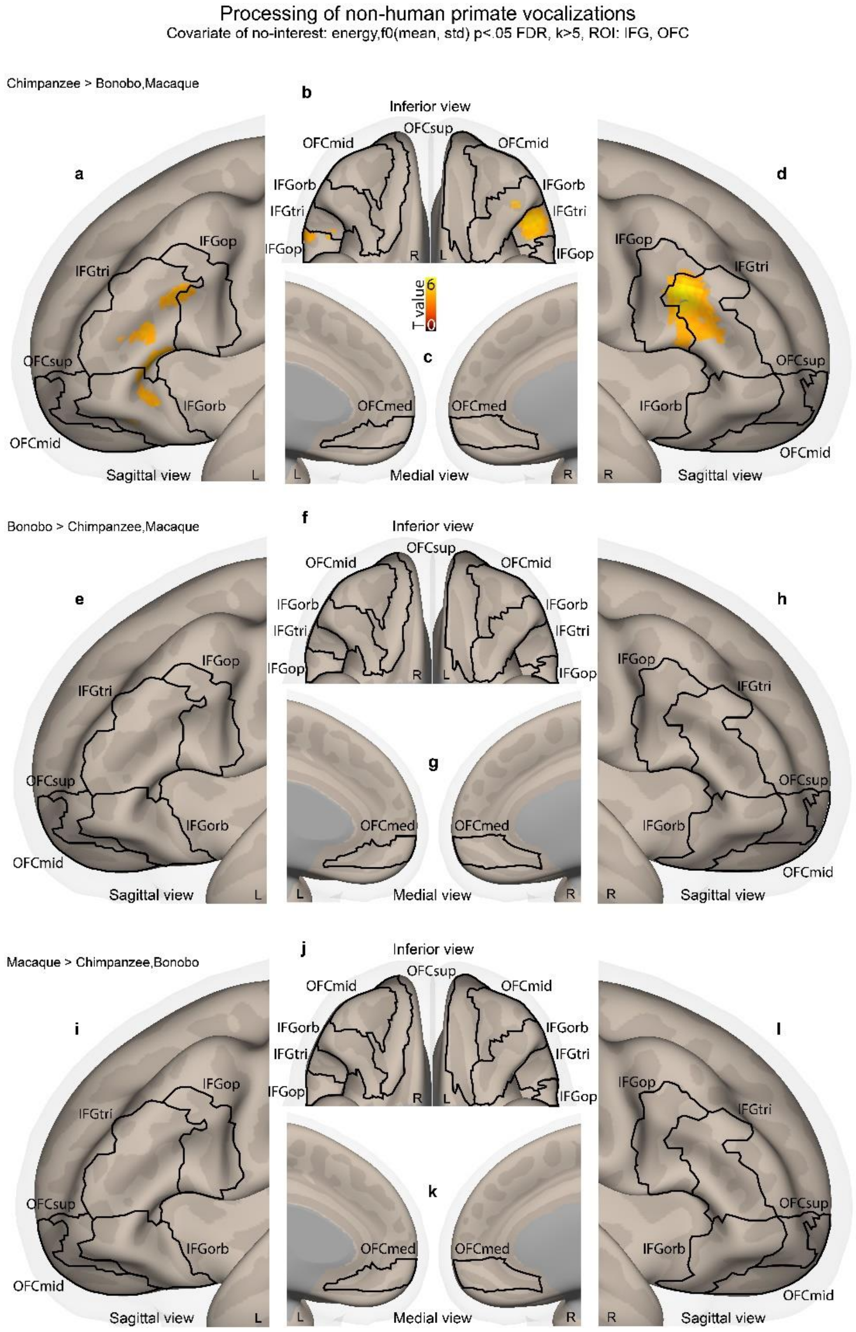

Fig.S7: Brain activations of non-human primate vocalizations processing within the IFG and OFC. abcd: Processing for Chimpanzee > Bonobo, Macaque vocalizations. **efgh**: Bonobo > Chimpanzee, Macaque vocalizations processing. **ijkl**: Macaque > Chimpanzee, Bonobo vocalizations processing. Trial-level covariates of no interest included the mean and standard deviation of the vocalization energy and fundamental frequency. Colorbars illustrate t-value statistics. All activations thresholded at a voxelwise p<.05 FDR, k>5 voxels, masked by IFG and OFC regions of interest (k=9635 voxels in total). IFG: inferior frontal gyrus; tri: *pars triangularis*; op: *pars opercularis*; orb: *pars orbitalis*; OFC: orbitofrontal cortex; med: medial; mid: middle; sup: superior.

Supplementary Table 1: Results for each factor of the modelling of accuracy data using generalized linear mixed-effects.

Effect	Chi-square value	Degrees of freedom	*p*-value
Species	222.78	3	<.001***
Affective context	4.64	2	.099.
Species*Affective context	12.78	6	.047*
Mean of F0	0.14	1	.712
Std of F0	2.94	1	.087.
Mean of Energy	0.00	1	.995
Std of Energy	0.01	1	.937

F0: voice fundamental frequency; Energy: voice spectral energy; . p<.10, *p<.05, ***p<.001.

Supplementary Table 2: Results for each factor and each species of the modelling of confusion data using linear mixed-effects.

Effect	Chi-square value	Degrees of freedom	*p*-value
**Human confusion**			
Species	1.65	3	.648
Affective context	0.21	2	.090.
Species*Affective context	4.65	6	.589
Mean of F0	0.03	1	.870
Std of F0	0.72	1	.398
Mean of Energy	0.07	1	.788
Std of Energy	1.18	1	.277
**Chimpanzee confusion**			
Species	265.79	3	<.001***
Affective context	6.44	2	<.039*
Species*Affective context	8.38	6	.211
Mean of F0	0.02	1	.887
Std of F0	0.10	1	.748
Mean of Energy	0.37	1	.542
Std of Energy	0.34	1	.561
**Bonobo confusion**			
Species	107.96	3	<.001***
Affective context	7.62	2	.022*
Species*Affective context	11.00	6	.088.
Mean of F0	4.56	1	.033*
Std of F0	6.65	1	.009**
Mean of Energy	0.02	1	.874
Std of Energy	0.09	1	.763
**Macaque confusion**			
Species	469.68	3	<.001***
Affective context	2.25	2	.325
Species*Affective context	6.27	6	.393
Mean of F0	4.96	1	.025*
Std of F0	4.38	1	.036*
Mean of Energy	1.15	1	.284
Std of Energy	0.95	1	.329

F0: voice fundamental frequency; Energy: voice spectral energy; . $p<.10$, *$p<.05$, **$p<.01$, ***$p<.001$.

Supplementary Table 3: Peak MNI coordinates of the Species factor for Human voice processing. (voxel-wise *p*<.05 FDR, k>5 voxels, wholebrain)

**Human voice**						
**Region**	**Hemisphere**	**MNI x**	**MNI y**	**MNI z**	**T value**	**Voxels**
STG_{ant}	L	-58	-12	-4	16.23	21471
STG_{post}	*L*	*-64*	*-40*	*14*	*12.23*	
STG_{mid}	*L*	*-54*	*-20*	*2*	*11.91*	
STG_{post}	R	54	-32	2	15.97	12070
STG_{mid}	*R*	*60*	*-14*	*2*	*14.29*	
STG_{ant}	*R*	*58*	*-6*	*-10*	*13.90*	
Cerebellum VIIIa	L	-24	-62	-54	9.32	674
Cerebellum VIIIa	R	24	-62	-54	6.29	435

STG: superior temporal gyrus; ant: anterior part; mid: middle part; post: posterior part.

Supplementary Table 4: Peak MNI coordinates of the correlates and anti-correlates of the probability of correctly classifying each species (voxel-wise p<.05 FDR, k>5 voxels, masked by bilateral IFG and OFC)

**Correlates**						
**Region**	**Hemisphere**	**MNI x**	**MNI y**	**MNI z**	**T value**	**Voxels**
OFC_{med}	L	-8	46	-6	4.15	227
IFG_{tri}	R	54	36	-2	3.36	16
OFC_{med}	L	-30	16	-22	3.33	6
IFG_{op}	R	60	16	10	3.12	13
**Anti-correlates**						
**Region**	**Hemisphere**	**MNI x**	**MNI y**	**MNI z**	**T value**	**Voxels**
IFG_{tri}	L	-46	38	8	8.15	1919
IFG_{tri}	R	36	28	12	7.22	403
IFG_{op}	R	40	4	26	4.56	53
IFG_{orb}	R	26	34	-8	4.54	53
IFG_{orb}	R	24	42	-12	3.41	5
OFC_{sup}	L	-40	54	-2	3.18	5

IFG: inferior frontal gyrus; OFC: orbitofrontal cortex; tri: *pars triangularis*; med: medial; op: *pars opercularis*; orb: *pars orbitalis*; sup: superior.

Supplementary Table 5: Peak MNI coordinates of conjunction analyses for non-human primate vocalization processing and correct categorization as compared to human voice (voxel-wise *p*<.05 FDR, k>5 voxels, masked by bilateral IFG and OFC)

**Conjunction: [Chimpanzee > Human] > [Bonobo > Human] > [Macaque > Human] (processing)**						
**Region**	**Hemisphere**	**MNI x**	**MNI y**	**MNI z**	**T value**	**Voxels**
IFG_{op}	L	-46	4	28	6.62	1337
IFG_{tri}	R	40	32	14	5.53	139
IFG_{op}	R	44	4	26	4.29	29
IFG_{orb}	L	-28	34	-12	4.17	22
IFG_{tri}	R	48	34	24	3.50	7
**Conjunction: [Chimpanzee > Human] > [Bonobo > Human] > [Macaque > Human] (correct responses)**						
**Region**	**Hemisphere**	**MNI x**	**MNI y**	**MNI z**	**T value**	**Voxels**
IFG_{tri}	L	-36	28	16	4.76	89

IFG: inferior frontal gyrus; tri: *pars triangularis*; op: *pars opercularis*; orb: *pars orbitalis*.
